# Distal Tibial Bone Properties and Bone Stress Injury Risk in Young Men Undergoing Arduous Physical Training

**DOI:** 10.1007/s00223-023-01111-1

**Published:** 2023-07-23

**Authors:** Katharine Eastman, Thomas J. O’Leary, Alexander Carswell, Neil Walsh, Rachel Izard, William Fraser, Julie Greeves

**Affiliations:** 1 Norwich Medical School, University of East Anglia, Norwich, UK; 2 Army Health and Performance Research, Army Headquarters, Andover, UK; 3 Division of Surgery and Interventional Science, UCL, London, UK; 4 School of Sport and Exercise Sciences, Liverpool John Moores University, Liverpool, UK; 5 Science and Technology Commissioning, Defence Science and Technology, Porton Down, Salisbury, UK; 6 Departments of Endocrinology and Clinical Biochemistry, Norfolk and Norwich University Hospitals, Norwich, UK; 7 HQ DPHC, Coltman House, DMS Whittington, Lichfield, WS14 9PY UK

**Keywords:** Bone stress injury, Dual x-ray absorptiometry, High-resolution peripheral quantitative computed tomography, Bone health, Training

## Abstract

Trabecular microarchitecture contributes to bone strength, but its role in bone stress injury (BSI) risk in young healthy adults is unclear. Tibial volumetric BMD (vBMD), geometry, and microarchitecture, whole-body areal BMD, lean and fat mass, biochemical markers of bone metabolism, aerobic fitness, and muscle strength and power were measured in 201 British Army male infantry recruits (age 20.7 [4.3] years, BMI 24.0 ± 2.7 kg·m^2^) in week one of basic training. Tibial scans were performed at the ultra-distal site, 22.5 mm from the distal endplate of the non-dominant leg using High Resolution Peripheral Quantitative Computed Tomography (XtremeCT, Scanco Medical AG, Switzerland). Binary logistic regression analysis was performed to identify associations with lower body BSI confirmed by MRI. 20 recruits (10.0%) were diagnosed with a lower body BSI. Pre-injured participants had lower cortical area, stiffness and estimated failure load (p = 0.029, 0.012 and 0.011 respectively) but tibial vBMD, geometry, and microarchitecture were not associated with BSI incidence when controlling for age, total body mass, lean body mass, height, total 25(OH)D, 2.4-km run time, peak power output and maximum dynamic lift strength. Infantry Regiment (OR 9.3 [95%CI, 2.6, 33.4]) Parachute *versus* Line Infantry, (p ≤ 0.001) and 2.4-km best effort run time (1.06 [95%CI, 1.02, 1.10], p < 0.033) were significant predictors. Intrinsic risk factors, including ultradistal tibial density, geometry, and microarchitecture, were not associated with lower body BSI during arduous infantry training. The ninefold increased risk of BSI in the Parachute Regiment compared with Line Infantry suggests that injury propensity is primarily a function of training load and risk factors are population-specific.

**Supplementary Information:**

The online version contains supplementary material available at 10.1007/s00223-023-01111-1.

## Introduction

Bone stress injuries (BSIs) at weight-bearing sites are common in military training, particularly at the tibia [1–3]. Repetitive loading causes fatigue damage, whereby targeted bone remodeling temporarily increases cortical porosity [4]. This temporary porosity weakens bone and, without sufficient recovery, can act as a site of concentrated stress leading to a BSI [4]. Bone stress injuries range from (i) periostitis; to (ii) periosteal, endosteal and bone tissue oedema; to (iii) partial or complete stress fracture [5]. The resistance of the tibia to bone stress injury is determined by bone strength and toughness, which, in turn is underpinned by structure (size, shape, and microarchitecture) and material properties [6]. The distal tibial microarchitecture distributes mechanical stress and joint forces to the cortex and is a determinant of bone strength however little is known about how distal tibial microarchitecture contributes to BSI risk.

Prospective and retrospective studies using peripheral quantitative computed tomography (pQCT) have reported lower total bone cross sectional area, cortical area, and estimated bone strength of the tibia in athletes and military recruits with stress fractures compared with uninjured controls [7–9]; some studies report no significant associations between pQCT outcomes and stress fracture [8, 9]. A wider bone increases resistance to bending forces by placing the cortex further away from the neutral axis [10]. This biomechanical advantage increases resistance to fatigue damage [10]. The low resolution of pQCT fails to detect the bone microstructure. Bone microstructure, including trabecular spacing, thickness and separation and cortical porosity, are important contributors to bone strength and may offer further insight into BSI risk [3, 11–14].

Post-injury cross-sectional studies using high-resolution pQCT (HRpQCT) have demonstrated lower cortical vBMD, trabecular number, trabecular thickness, and a more *inhomogenous* trabecular network at the ultra-distal site of the tibia (non-dominant side) in soldiers with stress fractures compared with uninjured controls [15]; lower trabecular vBMD and trabecular thickness at both ultra-distal and distal tibial sites [16, 17]; and, smaller cortical cross sectional area without an increase in cortical porosity [18] at the tibia in civilian stress fracture cases compared with uninjured controls. No study has prospectively examined pre-injury bone microstructure and BSI risk.

This study used HRpQCT to investigate pre-injury ultra-distal tibial microarchitecture as a predictor of lower body BSI (including BSI to the pelvic girdle, sacrum, coccyx and lower limb) in men during military training. The military training environment is a unique opportunity to prospectively study skeletal risk factors during a controlled model of arduous training with a high incidence of BSI. Infantry training is more arduous than other basic military training courses, with an incidence of stress fracture at 64 per 1000 [1, 19]. We hypothesised that trabecular microarchitecture and estimated mechanical strength would be associated with lower body BSI sustained during basic military training.

## Material and Methods

### Participants

**Fig. 1:**
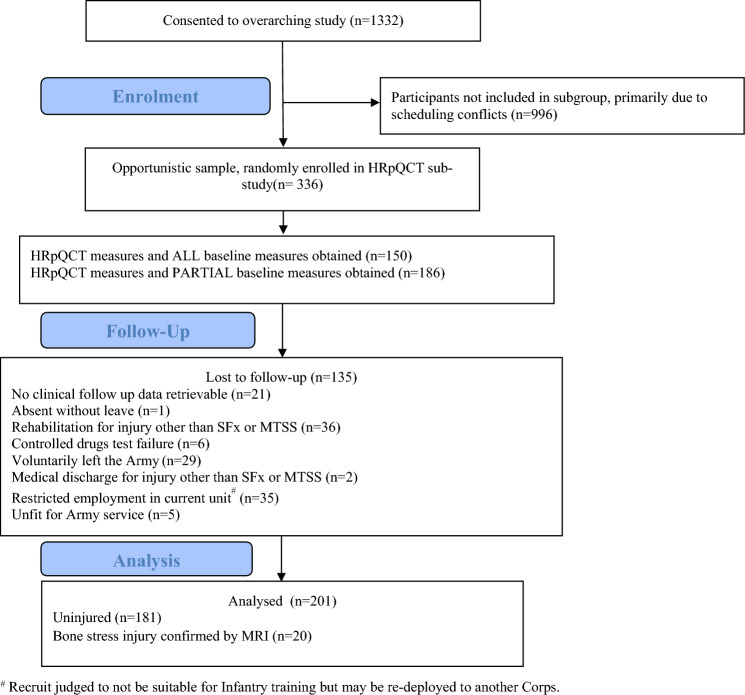
A modified STROBE diagram detailing participation in the study. ^#^ Recruit judged to not be suitable for Infantry training but may be re-deployed to another Corps

Male British Army infantry recruits (n = 1332) at the Infantry Training Centre, Catterick, UK, were assessed for eligibility as part of a larger study examining Army injury risk factors, with a convenience sample of 336 participants invited for HRpQCT measurements (Fig. 1). Participants were recruited during week 1 of basic military training between April 2014 and June 2016. All participants had passed a physician-screened medical assessment during week 1 of training and were injury free at baseline. The nature and purpose of the study were fully explained in writing and verbally to each participant, and all participants provided written informed consent. Ethical approval was obtained from the UK Ministry of Defence Research Ethics Committee (MODREC/165). All study procedures were conducted in accordance with the Declaration of Helsinki (2013).

### Experimental Protocol

Participants were undertaking either the Line Infantry 26-week or the Parachute Regiment 28-week training courses. Both courses are divided into two Phases: Phase One (14 weeks) teaches basic military skills and Phase Two (12 or 14 weeks) is infantry-specific combat training. The training courses include: endurance training, typically involving running in groups; load carriage; circuit training, consisting of high-repetition, low force exercises using all major muscle groups; agility-based gymnasium work using benches and ropes; assault courses; military drill, and; military field exercises [20]. Marching with various loads while on military exercise and military drill was also undertaken. Energy expenditures can exceed 4500 kcal/day with body mass losses reaching 14.4 kg; the greatest losses are observed in recruits of the Parachute Regiment who complete the most physically arduous training [13]. At the time of data collection, higher physical standards were required by recruits to pass Parachute Regiment training, who, as an example, were required to march 16.1 km in 110 min (8.9 km·h^−1^) carrying a minimum of 23 kg compared with the Line Infantry who are required to march 12.9 km in 120 min (6.4 km·h^−1^) carrying 25 kg.

All measurements were obtained in week one of the course, after the medical assessment and before starting physical training. Participants in this sub-analysis had a HRpQCT scan of the ultra-distal tibia for the measurement of bone density, geometry, microarchitecture, and estimated mechanical strength. Whole-body areal BMD (aBMD), lean mass, and fat mass were measured by dual energy x-ray absorptiometry (DXA). A venous blood sample was obtained for the assessment of biochemical markers of bone metabolism. Aerobic fitness, and muscle strength and power, were measured with field-based tests of physical fitness. Self-reported demographic and lifestyle characteristics were recorded by questionnaire.

### Tibial Volumetric Bone Mineral Density, Geometry, and Microarchitecture

Three-dimensional HRpQCT scans (XtremeCT; Scanco Medical AG, Bassersdorf, Switzerland) were performed at the ultra-distal site of the non-dominant tibia. Limb dominance was defined as the leg primarily used to kick a ball [21]. Daily quality control checks with a standardised control phantom were conducted according to manufacturer guidelines. The tested leg was immobilised in a carbon-fibre cast for scanning. The reference line was set manually at the distal end plate, with the region of interest defined using the anteroposterior scout view. A three-dimensional representation of 9.02 mm of bone length was taken from a total of 110 slices with an isotropic voxel size of 82 μm. The first slice was taken 22.5 mm from the distal end plate. Scans were motion graded [1–3] according to manufacturer guidelines by a single operator, with grade 3 scans repeated once. The methods used to process the data have been previously described [22].

The standard evaluation procedure provided by the manufacturer (software version 6.0) was used to derive the following outcome variables: total, trabecular, and cortical vBMD (mg HA·cm^3^); trabecular bone volume (%); trabecular area (mm^2^); trabecular number (1•mm); trabecular thickness (mm); trabecular spacing (mm); cortical area (mm^2^); cortical thickness (mm); cortical perimeter (mm); cortical pore diameter (mm) and cortical porosity (%). Finite element analysis was performed using manufacturer software, as described previously [23] to calculate biomechanical properties stiffness (kN mm) and failure load (kN) under uniaxial compression. A single investigator performed all contouring to ensure consistency. The coefficient of variation (CV) for parameters in our laboratory ranges from 0.1 to 3.2% for geometry, density, and microarchitecture, and from 9.5% to 10.0 for cortical porosity (unpublished data).

### Areal Bone Mineral Density, Total Fat Mass, Lean Body Mass

A whole-body DXA scan (Lunar iDXA™, GE Healthcare, UK.) was performed with participants wearing only underwear and lying supine on the scanning table. Velcro straps were placed around the knees and ankles to minimise movement. Whole-body aBMD, fat mass, and lean mass, and leg aBMD were analysed from the scan. Daily quality control checks with a standardised control phantom were conducted according to manufacturer guidelines. The CV for leg aBMD in our laboratory is 0.6% (unpublished data).

### Blood Collection and Handling

Whole blood samples were collected by venepuncture from an antecubital vein into one serum vacutainer and one EDTA vacutainer (Becton Dickinson, Oxford, UK). Whole blood in serum vacutainers was left to clot at room temperature for 60 min and then spun at 1500 g for 10 min at 4 °C. EDTA (plasma) samples were centrifuged immediately at 1500 g for 10 min at 4 °C. Serum and plasma were aliquoted into universal tubes, and immediately frozen at − 80 °C for later analysis.

### Biochemical Markers of Bone Metabolism

Serum samples were analysed for albumin-adjusted calcium and iron (COBAS c601, Roche, USA) and total 25-hydroxyvitamin D (25(OH)D) by high-performance liquid chromatography tandem mass spectrometry (LC–MS/MS) (Micromass Quattro Ultima Pt electrospray ionisation tandem mass spectrometer, Waters Corp., Milford,MA, USA) using MassLynx version 4.1 and QuanLynx software (Waters Corp., Milford, MA, USA) [24]. EDTA plasma samples were analysed for parathyroid hormone (PTH), procollagen type 1 N-terminal propeptide (P1NP), and beta C-terminal telopeptide (βCTX) by electro-chemical luminescence immunoassay (ELICIA) on the COBAS c601 (Roche Diagnostics, Mannheim, Germany) platform. All biochemical analyses were undertaken by the Clinical Pathology Accredited Bio-Analytical Facility at the University of East Anglia. All analytical processes meet the requirements set by external national quality assurance schemes including the National External Quality Assurance Scheme and Vitamin D External Quality Assessment Scheme.

### Anthropometric Data

Height and body mass were measured without shoes and with light clothing using a stadiometer and a digital platform scale (Seca, Hamburg, Germany).

### Physical Performance

Aerobic fitness was determined from a best effort 2.4 km run time, performed on a standardised course during routine testing at the Infantry Training Centre, Catterick. Maximum dynamic lift strength was assessed using an incremental lift machine that simulates the power clean weight-lifting movement, as described previously [25]. The device consisted of a vertically moving carriage with handgrips 0.3 m above the ground. Participants started the incremental lifts with a mass of 20 kg, with the weight lifted to a point where the handgrips were 1.45 m from the ground replicating the floor height of an Army four tonne truck. With each successful lift, the mass was increased by 5 kg with 1 min rest between attempts. The test was terminated when participants failed to lift the weight on their second attempt, with the maximum weight successfully lifted recorded. Peak power output was measured using a maximum vertical jump height determined using a digital jump meter (Takei Scientific Instruments, Tokyo, Japan) as described [25]. Participants were instructed to jump as high as possible three times. If an increase in jump height occurred across three repetitions, indicating a learning effect, a fourth jump was performed. Maximum vertical jump height of the attempts was recorded as the highest score achieved. Peak power output was calculated from maximum jump height and body mass using a validated equation reflecting instantaneous power output [26]: Peak power output (W) = (51.9 × jump height (cm)) + (48.9 × body mass (kg))–2007.

### Bone Stress Injury Diagnosis

Lower body BSI diagnoses (metatarsal, cuneiform, calcaneus, tibia, pubic rami, femur and neck of femur) were retrieved from participants’ individual medical record. BSI diagnoses were determined by magnetic resonance imaging (MRI) and graded according to the Fredericson scale [27, 28].

### Statistical Analyses

Data were analysed using SPSS (v. 25, SPSS Inc., IBM, USA). All data were initially checked for normality. All outcomes were compared between male recruits who sustained a BSI and those who did not, using chi-squared tests or independent sample t-tests (or Mann–Whitney U tests for non-parametric data) as appropriate. Forced entry binary logistic regression was used to determine risk factors for BSI. Model one included training regiment, age at start of basic military training, total body mass, lean body mass, height, leg aBMD (average of both legs), and circulating total 25(OH)D, PTH, adjusted calcium, iron, P1NP, and βCTX. Model two included the variables in model one *plus* physical performance (2.4 km run time, maximum dynamic lift strength, and peak power output). Model three included all the variables in model two *plus* distal tibia HRpQCT measures. All variables considered for inclusion were checked for collinearity using variance inflation factors; variables scores ≥ 10 were removed. Statistical significance was accepted at p < 0.05. This is an exploratory analysis and a power calculation was not performed.

## Results

Participants (n = 1332) provided consent and 336 participants were selected at random for HRpQCT scans. 135 participants were lost to follow-up because they did not complete training for a reason other than suffering a BSI. 201 participants were included in the analyses; 181 were uninjured (no musculoskeletal injury and completed the training course) and 20 sustained a BSI (Fig. 1). The analysis included all available baseline data.

### Bone Stress Injury Incidence

Twenty participants were diagnosed with a BSI by MRI representing 10.0% of the population at risk. slightly higher than the previously reported rate of 6.3% [19]. BSIs were graded between 1 and 4b on the Fredericson scale [27] and the median week of presentation with an injury was 6.5 [IQR 8.0] weeks after commencing training. The incidence of BSI was higher in the Parachute Regiment (*n* = 14, 21.9% of the population) compared with Line Infantry (*n* = 6, 4.4% of the population) (p < 0.001). Anatomical sites of BSI are listed in Table 1 and a full description of all injuries is contained within supplementary material 1. No participant presented with multiple BSIs.

**Table 1:** Anatomical sites of bone stress injuries (BSIs)

Anatomical site	Number of BSIs (%)	Number of BSIs in parachute regiment recruits* (%)	Number of BSIs in line Infantry recruits (%)
Tibia	7 (35.0)	7 (50.0)	0 (0.0)
Calcaneus	4 (20.0)	4 (28.6)	0 (0.0)
Metatarsal	3 (15.0)	1 (7.1)	2 (33.3)
Cuneiform	2 (10.0)	0 (0.0)	2 (33.3)
Pubic Rami	1 (5.0)	1 (7.1)	0 (0.0)
Femur	1 (5.0)	0 (0.0)	1 (16.7)
Neck of Femur	1 (5.0)	1 (7.1)	0 (0.0)
Site not recorded	1 (5.0)	0 (0.0)	1 (16.7)

*Do not add up to 100% due to rounding

### Differences Between Bone Stress Injury Cases and Controls

Pre-injury demographics, body composition, physical performance, and biochemistry were not significantly different between non-BSI controls and BSI cases. Cortical area (p = 0.029), stiffness (p = 0.012), and estimated failure load (p = 0.011) were significantly lower in BSI cases compared with controls (Table 2).

**Table 2:** Participant characteristics by injury status

	Overall (n = 201)	Non-BSI (n = 181)	BSI (n = 20)	*p*
*Demographics*				
Regiment				
Line Infantry	137 (100.0%)	131 (95.6%)	6 (4.4%)	< 0.001*
Parachute	64 (100.0%)	50 (78.1.6%)	14 (21.9%)	
Alcohol Intake^a^				
Zero Intake	18 (100.0%)	17 (94.4%)	1 (5.6%)	0.651
Light (1–90 units per month)	124 (100.0%)	112 (90.3%)	12 (9.7%)	
Heavy (91–300 units per month)	47 (100.0%)	40 (85.1%)	7 (14.9%)	
Very Heavy (> 300 units per month)	1 (100.0%)	1 (100.0%)	0	
Smoking^b^				0.412
Never Smoked	76 (100.0%)	69 (39.4%)	7 (35.0%)	
Previous Smoker	54 (100.0%)	60 (34.3%)	5 (25.0%)	
Current Smoker	65 (100.0%)	46 (26.3%)	8 (40.0%)	
Ethnicity^c^				0.657
Asian	2 (100.0%)	2 (100.0%)	0	
Black	3 (100.0%)	3 (100.0%)	0	
Mixed	3 (100.0%)	2 (66.7%)	1 (33.3%)	
White	187 (100.0%)	168 (89.8%)	19 (10.2%)	
Other	1 (100.0%)	1 (100.0%)	0	
Age at Start of Basic Military Training (years)	20.7 [4.3]	20.7 [4.1]	22.9 [5.0]	0.264
*Body Composition and Physical Performance*				
Height (cm)	177.7 ± 6.2	177.7 ± 6.2	177.7 ± 5.7	0.486
Body Mass Index (kg/m^2^)	24.0 ± 2.7	24.1 ± 2.8	23.1 ± 2.0	0.112
Total Body Mass (kg)	76.0 ± 10.1	73.0 ± 8.6	76.3 ± 10.2	0.129
Lean Body Mass (kg)^e^	57.2 [6.8]	58.0 [7.0]	54.7 [4.1]	0.098
Leg aBMD (g∙cm^2^)^e^	1.36 ± 0.13	1.36 ± 0.13	1.34 ± 0.14	0.441
2.4 km run time (s)^f^	597 [91]	598 [89]	568 [86]	0.130
Maximum Dynamic Lift (kg)^g^	73 [20]	75 [20]	70 [19]	0.414
Peak Power Output (W)^h^	3940 ± 540	3969 ± 536	3731 ± 532	0.083
*Biochemistry*				
Total 25(OH)D (nmol·L^−1^)	58.3 [43.2]	57.5 [42.9]	61.0 [49.3]	0.389
PTH (pmol·L^−1^)^d^	3.50 [1.51]	3.47 [1.62]	3.77 [1.39]	0.168
Adjusted Calcium (mmol·L^−1^)	2.39 [0.09]	2.39 [0.09]	2.40 [0.12]	0.390
Iron (µmol·L^−1^)	20.3 [8.5]	20.5 [8.5]	19.5 [11.7]	0.327
P1NP (µg∙L)^d^	92.8 [45.2]	92.8 [45.2]	97.0 [48.7]	0.650
CTX (µg∙L)^d^	0.47 [0.22]	0.47 [0.22]	0.43 [0.20]	0.175
*Bone Parameters*				
Total vBMD (mg HA·cm^3^)	350 ± 50	351 ± 50	338 ± 48.0	0.280
Trabecular vBMD (mg HA·cm^3^)	231 ± 33	232 ± 32	223 ± 37	0.206
Total Area (mm^2^)	840 [188]	846 [194]	791 [165]	0.160
Trabecular Area (mm^2^)	690 [205]	794 [207]	647 [172]	0.364
Cortical vBMD (mg HA·cm^3^)	889 [47]	891 [29]	896 [60]	0.903
Trabecular Number (1·mm)	2.20 ± 0.29	2.21 ± 0.28	2.12 ± 0.35	0.192
Trabecular Thickness (mm)	0.09 ± 0.01	0.09 ± 0.01	0.09 ± 0.01	0.946
Trabecular Spacing (mm)	0.364 [0.073]	0.361 [0.069]	0.369 [0.116]	0.237
Cortical Area (mm^2^)	140 ± 21	141 ± 21	130 ± 17	0.029*
Cortical Perimeter (mm)	113.9 [12.7]	114.2 [12.7]	110.3 [11.0]	0.096
Cortical Porosity (%)	5 [2]	5 [2]	4 [2]	0.217
Cortical Thickness (mm)	1.32 ± 0.24	1.33 ± 0.25	1.26 ± 0.19	0.205
Cortical Pore Diameter (mm)	0.161 [0.022]	0.160 [0.021]	0.167 [0.026]	0.688
Stiffness (kN∙mm)	284.0 ± 41.18	286.4 ± 40.71	262.08 ± 39.9	0.012*
Estimated Failure Load (kN)	-14.2 ± 2.0	-14.3 ± 2.0	-13.1 ± 1.9	0.011*

Categorical data are presented as total (percentage) with p-values calculated by Chi-Squared. Continuous data are mean ± SD with p-values from independent t-tests or median [IQR] with p-values from Mann–Whitney U Tests

^a^overall n = 190, non-BSI n = 170

^b^overall n = 195, non-BSI n = 175

^c^overall n = 196, non-BSI n = 176

^d^overall n = 200, non-BSI n = 180

^e^overall n = 192, non-BSI n = 173, BSI n = 19

^f^overall n = 160, non-BSI n = 143, BSI n = 17

^g^overall n = 144, non-BSI n = 128, BSI n = 16

^h^overall n = 149, non-BSI n = 131, BSI n = 18

### Risk Factors for Bone Stress Injuries

In Model 1, training regiment was the only variable associated with BSI incidence ((OR 9.3 [95%CI, 2.6, 33.4]) Parachute *versus* Line Infantry, p ≤ 0.001) when training course, age at start of military training, total body mass, lean body mass, height, leg aBMD, and total 25(OH)D were included. Adding physical performance (2.4-km run time, maximum dynamic lift strength, and peak power output) (Model 2) identified both training course and 2.4-km run time as associated with BSI incidence; for every 1 s increase in run time, there was a 5.5% increase in BSI risk (1.06 [95%CI, 1.02, 1.10), p < 0.04). Adding total area, cortical vBMD, trabecular thickness and cortical pore diameter (Model 3), did not change these findings; training course and 2.4-km run time remained the only associated factors (Table 3). Total vBMD, trabecular vBMD, trabecular area, trabecular spacing, cortical area, cortical vBMD, cortical perimeter, cortical porosity, cortical thickness, stiffness, and failure load were removed due to collinearity.

**Table 3:** Factors associated with BSI incidence in British Army infantry recruits

Predictor variable	Co-efficient (95% CI)	*p*
Regiment †	748.90* (6.85 to 81,866.10)	**0.006**
Age at Start of Military Training	0.94 (0.54 to 1.62)	0.813
Total Body Mass	0.83 (0.54 to 1.16)	0.282
Lean Body Mass	1.00 (1.00 to 1.00)	0.053
Height	1.05 (0.83 to 1.31)	0.698
Leg aBMD	0.98 (0.96 to 1.01)	0.288
Total 25(OH)D	1.03 (0.98 to 1.07)	0.223
2.4-km Run Time	1.05 (1.01 to 1.09)	**0.020**
Peak Power Output	1.00 (0.99 to 1.00)	0.196
Maximum Dynamic Lift Strength	1.00 (0.90 to 1.10)	0.721
Total Area	0.99 (0.97 to 1.01)	0.198
Cortical vBMD	1.01 (0.98 to 1.05)	0.487
Trabecular Thickness	2.08 (0.00 to 6.00 × 10^65^)	0.282
Cortical Pore Diameter	0.00 (0.00 to 3.35 × 10^21^)	0.368

Bold values indicate significant result

†Parachute Regiment vs Line Infantry (reference group)

*Odds Ratio

### Description of Parachute Regiment Participants

Most injuries were in Parachute Regiment trainees so exploratory analysis was conducted in this sub-group to better understand which, if any, factors contributed to the risk of BSI. Baseline demographics of the participants undergoing Parachute Regiment training overall, and by BSI status, are shown in Table 4. Uninjured participants had significantly higher total lean mass (p = 0.025) and leg aBMD (p = 0.012) compared with BSI cases.

**Table 4:** Baseline demographics, alcohol intake, smoking status and ethnicity of participants undergoing parachute regiment training

	Overall (n = 64)	Non-BSI (n = 50)	BSI (n = 14)	*P*
*Demographics*				
Alcohol Intake				
Zero Intake	4 (6.3%)	4 (8%)	0	0.089
Light (1–90 units per month)	42 (65.6%)	35 (70%)	7 (50%)	
Heavy (91–300 units per month	18 (28.1%)	11 (22%)	07 (50%)	
Very Heavy (> 300 units per month)	0			
Smoking				
Never Smoked	31 (48.4%)	24 (48%)	7 (50%)	
Previous Smoker	28 (43.8%)	21 (42%)	7 (50%)	0.456
Current Smoker	5 (7.8%)	5 (10%)	0	
Ethnicity				
Asian	2 (3.1%)	2 (4%)	0	0.644
Black	1 (1.6%)	1 (2%)	0	
White	61 (95.3%)	47 (94%)	14 (93.3%)	
Age at Start of Military Training (years)	21.61 [4.86]	21.33 [4.76]	22.63 [5.29]	0.697
*Body Composition and Physical Performance*				
Height (cm)	177.3 ± 6.2	177.4 ± 6.5	176.9 ± 5.2	0.400
Body Mass Index (kg/m^2^)	24.0 ± 2.3	24.3 ± 2.4	23.0 ± 1.8	0.059
Total Body Mass (kg)	75.7 ± 9.5	72.2 ± 9.0	76.7 ± 9.5	0.123
Total Lean Mass (kg)^a^	59.1 ± 6.3	60.1 ± 6.6	55.6 ± 3.7	0.025*
Leg aBMD (g/cm^2^)^a^	1.39 [0.21]	1.41 [0.22]	1.27 [0.17]	0.012*
2.4-km Run Time (secs)^b^	551 [30]	547 [47]	552 [30]	0.296
Maximum Lift (kg)^c^	75.0 [15]	75 [18]	74 [15]	0.338
Peak Power Output (W)^d^	4005 ± 559	4081 ± 549	3790 ± 555	0.122
*Biochemistry*				
Total 25(OH)D (nmol·L^−1^)	69.0 ± 25.9	68.6 ± 25.9	70.5 ± 26.8	0.565
PTH (pmol·L^−1^)^e^	3.83 ± 1.22	3.84 ± 1.32	3.82 ± 0.81	0.964
Adjusted Calcium (mmol·L^−1^)	2.37 ± 0.07	2.36 ± 0.07	2.38 ± 0.07	0.264
Iron (µmol·L^−1^)	20.5 ± 7.4	20.7 ± 7.3	20.1 ± 7.9	0.792
P1NP (µg/L)^f^	93.5 [51.8]	93.5 [51.2]	96.6 [55.6]	0.791
CTX (µg/L)^f^	0.45 [0.19]	0.45 [0.18]	0.46 [0.25]	0.710
*Bone parameters*				
Total vBMD (mg HA·cm^3^)	353 ± 52	3547 ± 53	349 ± 50.0	0.744
Trabecular vBMD (mg HA•cm^3^)	232 ± 38	233 ± 38	229 ± 38	0.691
Total Area (mm^2^)	850 ± 127	8638 ± 129	802 ± 107	0.114
Trabecular Area (mm^2^)	697 ± 128	708 ± 131	660 ± 111	0.212
Cortical vBMD (mg HA·cm^3^)	888 [52]	888 [47]	893 [64]	0.826
Trabecular Number (1·mm)	2.13 ± 0.40	2.25 ± 0.30	2.13 ± 0.40	0.227
Trabecular Thickness (mm)	0.087 ± 0.013	0.087 ± 0.013	0.090 ± 0.010	0.351
Trabecular Spacing (mm)	0.36 [0.08]	0.353 [0.73]	0.37 [0.10]	0.363
Cortical Area (mm^2^)	143 ± 20	145 ± 20	134 ± 17	0.075
Cortical Perimeter (mm)	114.1 [13.3]	115.9 [12.6]	109.7 [11.2]	0.077
Cortical Porosity (%)	5 ± 2	5 ± 2	5 ± 2	0.645
Cortical Thickness (mm)	1.35 ± 0.22	1.36 ± 0.22	1.31 ± 0.19	0.401
Cortical Pore Diameter (mm)	0.161 [0.019]	0.159 [0.019]	0.169 [0.019]	0.121
Stiffness (kN∙mm)	283.72 ± 47.76	288.58 ± 48.86	266.37 ± 40.55	0.125
Estimated Failure Load (kN)	−14.19 ± 2.31	−14.44 ± 2.38	−13.31 ± 1.88	0.108

Categorical data are presented as total (percentage), p-values calculated by Chi Squared. Continuous data are mean ± SD, p-value calculated by independent samples t-tests or median [IQR], p-value calculated by Mann–Whitney U Test

^a^overall *n* = 58, non-BSI *n* = 45, BSI *n* = 13

^b^overall *n* = 52, non-BSI *n* = 40, BSI *n* = 13

^c^overall *n* = 43, non-BSI *n* = 33, BSI *n* = 10

^d^overall *n* = 46, non-BSI* n* = 34, BSI *n* = 12

^e^overall *n* = 53, non-BSI *n* = 49

^f^overall *n* = 63, non-BSI *n* = 49

## Discussion

BSIs are a financial and healthcare burden for defence, yet effective strategies to mitigate such injuries remain poorly understood. BSIs are caused by sudden changes in repetitive, high impact training loads. Basic military training is physically and mentally arduous [12, 13, 29], increases bone strength (tibial density, geometry, and microarchitecture) [11, 21, 30–33], and results in a high incidence of lower limb BSIs [1]. During this study 10.0% of the population at risk were diagnosed with a BSI, slightly higher than the previously reported rate of 6.3% [19].

Trabecular microarchitecture at the ultra-distal tibia was not associated with BSI incidence in this group of healthy male young adults undergoing arduous military training. Neither volumetric density, geometry and estimated bone strength of the ultra-distal tibia, nor biochemical markers of bone metabolism, were associated with lower body BSIs in the same population. We have demonstrated in both case–control comparisons and binary logistic regression that training Regiment is an important determinant of BSI; the incidence of injuries was higher in the Parachute Regiment compared with the Line Infantry during basic training. Physical fitness levels, including 2.4-km run time and maximum dynamic lift strength, were higher in the Parachute Regiment, but controlled for in this study.

### Trabecular Microarchitecture

Trabecular microarchitecture was measured at the ultra-distal tibia. The dense trabecular network has an anisotropic distribution aligned parallel to the mechanical stress axis and is important for absorbing and distributing mechanical stresses to the cortex [34]. Therefore, trabecular microarchitecture acts to resist compressive forces and is an important contributor to bone strength [35–38]. Our data show that trabecular microarchitecture was not associated with BSI incidence in this cohort of men during basic military training. A cross sectional study in a male military population reported lower cortical vBMD, trabecular number, trabecular thickness, and greater *inhomogenous* trabecular network in the tibial bone stress injury group compared with uninjured controls [15]. Bone microarchitecture did not play a role in BSI risk in our cohort of physically fit male infantry recruits, possibly due to mechanical adaptation experienced before entry to training; this supposition is supported by the observations that trabecular microarchitecture did not change in response to the same training course from a larger cohort in the same study [30]. In women, increased trabecular thickness and number, decreased separation, and increased *estimated* strength, during basic military training have been observed in as little as 8 weeks [21, 31]; and, case–control studies reported lower trabecular vBMD and trabecular thickness [16] at the ultra-distal site with no difference in vBMD, cortical or trabecular measures at the ultra-distal site [17] in BSI cases compared with controls. The contribution of trabecular microarchitecture to BSI risk may depend on sex or physical fitness status influencing mechanical adaptation.

### Bone Geometry

Cortical area was lower at baseline in recruits who sustained a BSI compared with uninjured controls in bivariate analysis, but no bone characteristics were associated with BSI risk in our binary logistic regressions. Retrospective HRpQCT studies in female athletes with a history of lower limb stress fractures reported lower cortical area at the distal tibia than those with no history of stress fracture injury [17], possibly due to the unloading, or reduction in training load as a result of the BSI or its symptoms. A greater cortical area increases strength under axial loading as the tibial cortex is further from the neutral axis and the resistance to bending is increased [6], which also likely explains the lower estimated failure load in BSI cases in our study. Another study in female athletes also reported lower stiffness and failure load in those with a history of multiple stress fracture (≥ 2) compared with those with < 2 fractures [16]. Therefore, greater cortical area is likely protective against tibial BSI as most tibial BSIs occur at the distal third of the tibia where the proportion of cortical bone and bending stresses are greatest [6]. Popp et al*.* reviewed skeletal properties at multiple tibial sites and reported differences in bone properties at the proximal third (66%)—more slender tibias, lower stress strain indices, lower section moduli and smaller total cross sectional and cortical areas were observed in runners with bone stress injury compared with uninjured runners [7]. Izard et al*.* reported significant increases in cortical area, thickness and bone strength at the 38% site after undertaking 10 weeks of military training, which is the site of highest mechanical stresses during locomotion [11] and these more proximal measures of geometry may be better predictors of BSI risk [39].

### Training Load

Training Regiment and 2.4-km run time were the only factors associated with BSI incidence in this study, with the Parachute Regiment having a ninefold increased risk of BSI when controlling for other factors. The Parachute Regiment have faster 2.4-km run time entry standards than the Line Infantry, yet they suffered a higher BSI incidence. Accordingly, adding 2.4-km run time to the model markedly increased the odds ratio for the Parachute Regiment, but these coefficients should be interpreted with caution because the confidence intervals were wide and so the exact estimates are unclear. The small co-efficient associated with 2.4-km run time is likely a function of the combination of Parachute Regiment and Lines recruits. The higher entry fitness standards required for the Parachute Regiment are necessary to achieve their superior in-service role-related fitness standards; these occupational demands are reflected in a higher training intensity and volume of basic training that accentuates skeletal loading and, probably, increases propensity to BSI.

Slower 2.4-km run time has been consistently associated with increased risk of BSI injury in female recruits [40, 41]. Slower run times suggest that recruits enter training with a lower level of aerobic fitness and are exposed to greater training demands when training alongside aerobically fitter peers. The higher training demands of the Parachute Regiment compared with other courses [12, 18) outweighs the risk of BSI from demographic, lifestyle behaviours and bone phenotype [42]. The physical demands of the Parachute Regiment training have been previously described in full [13, 20]. Both infantry populations in this study undertake a common military syllabus, but the training intensity and content is variable, based on the specific role recruits are being trained for. The loaded march standards provide an example of the higher physical standards that must be attained by recruits to pass Parachute Regiment training. Parachute Regiment recruits carry marginally less weight than the line infantry (23 kg vs 25 kg), however, they march at a faster pace for a longer distance (8.9 km·h^−1^ [total of 16.1 km in 110 min] *vs* 6.4 km·h^−1^ [total of 12.9 km in 120 min]). The pattern of injury, specifically pronounced tibia and calcaneus BSI in the Parachute Regiment, suggests increased ground reaction and muscle forces on limbs with increasing speed. In this study we found the training load required to carry load at faster speeds for longer distances is the most important determinant of BSI risk in men during physically arduous courses.

### Strengths and Limitations

All bone stress injuries included in this study were diagnosed by MRI, which is considered the gold standard method for their diagnosis. We included all lower body BSIs in this study, but the contribution of bone size and shape to BSI resistance might be localised.

The ultra-distal tibia was selected as the focus of this study due to the dense trabecular bone, however Popp et al. have previously identified that bone properties at the proximal (66% site) tibial site show the greatest difference between runners with bone stress injury compared with uninjured runners [7].

We did not record exercise levels prior to starting basic training, but anecdotally recruits increase their training volume in preparation for basic training, consistent with the peak of BSIs occurring in the early weeks of training [43]. Low levels of physical activity and uniaxial loading in high school are associated with an increased risk of multiple stress fractures [44]. The 2.4-km best effort run time was used as a surrogate measure of aerobic fitness prior to joining the military as recollection of physical activity in formative years was deemed to be unreliable in this participant cohort; but the 2.4-km best effort run time measurement may be influenced by physical preparation undertaken immediately prior to commencing initial training.

The sample size was small in this study with only 20 events (BSIs) recorded and so our ability to identify associated risk factors was limited when considered the number of events per variable. The p values in this study must also be interpreted with caution as no correction was applied and a large number of factors were compared between our injured and non-injured groups; our results may, therefore, be subject to type I error. Women were not included in this study because they were not then eligible for infantry training; but, since the changes in employment policy to include women in ground close contact roles, further studies are required to establish the role of trabecular microarchitecture as a predictor of BSI risk in women.

## Conclusions

Intrinsic risk factors, including ultradistal tibial density, geometry, and microarchictecture, were not associated with lower body BSI during arduous infantry training. The ninefold increased risk of BSIs in the Parachute Regiment compared with Line Infantry suggests that injury propensity is primarily a function of training load and risk factors are population-specific.

## Supplementary Information

Below is the link to the electronic supplementary material.

Supplementary file1 (DOCX 23 KB)

## Abbreviations

25(OH)D: 25-Hydroxyvitamin D
ACa: Albumin-adjusted calcium
βCTX: C-telopeptide cross-links of type 1 collagen
Ct.Area: Cortical area
Ct.Pm: Cortical perimeter
Ct.Po: Cortical porosity
Ct.Th: Cortical thickness
Ct.vBMD: Cortical volumetric bone mineral density
pQCT: Peripheral quantitative computed tomography
DXA: Dual energy X-ray absorptiometry
HRpQCT: High-resolution peripheral quantitative computed tomography
P1NP: Procollagen type 1 N-terminal propeptide
PTH: Parathyroid hormone
Tb.Area: Trabecular area
Tb.BV/TV: Trabecular bone volume fraction
Tb.N: Number of trabeculae
Tb.Sp: Trabecular separation
Tb.Th: Trabecular thickness
Tb.vBMD: Trabecular volumetric
BMD: Bone mineral density
vBMD: Volumetric bone mineral density
aBMD: Areal bone mineral density
MODREC: Ministry of Defence Research Ethics Committee
MRI: Magnetic resonance imaging
