# Supplementary material for: Distal Tibial Bone Properties and Bone Stress Injury Risk in Young Men Undergoing Arduous Physical Training
Source: Calcif Tissue Int. 2023 Jul 23;113(3):317–28. doi: 10.1007/s00223-023-01111-1 (PMC10449708; doi:10.1007/s00223-023-01111-1)
Supplement: Supplementary file 1 — Supplementary file1 (DOCX 23 KB) [file 223_2023_1111_MOESM1_ESM.docx]

Supplementary Material

Supplementary Table 1: Description of stress fracture injuries by stress fracture site, the grade of the injury on the Fredericson scale, week of presentation following starting military training, the type of training the recruit was undertaking and the phase of training the stress fracture occurred in.

| Injury No. | Site of Stress Fracture | Grade^a^ | Week of Presentation (Type of Training: Phase) |
| --- | --- | --- | --- |
| 1 | Metatarsal | 3 | 9 (L:1) |
| 2 | Cuneiform | 3 | 4 (L:1) |
| 3 | Cuneiform | 4b | 6 (L:1) |
| 4 | Calcaneus | 4b | 2 (P:1) |
| 5 | Calcaneus | 4b | 7 (P:1) |
| 6 | Metatarsal | 4b | 5 (P:1) |
| 7 | Calcaneus | 4b | 2 (P:1) |
| 8 | Calcaneus | 4b | 5 (P:1) |
| 9 | Metatarsal | Not Recorded | 19 (L:2) |
| 10 | Tibia | 1 | 6 (P:1) |
| 11 | Tibia | 2 | 10 (P:1) |
| 12 | Tibia | 2 | 9 (P:1) |
| 13 | Tibia | 2 | 13 (P:1) |
| 14 | Tibia | 2 | 2 (P:1) |
| 15 | Tibia | 2 | 21 (P:2) |
| 16 | Tibia | 4b | 21 (P:2) |
| 17 | Pubic Rami | 4a | 4 (P:1) |
| 18 | Femur | 4a | 6 (L:1) |
| 19 | Neck of Femur | 4 | 17 (P:2) |
| 20 | Site not recorded | 3 | 10 (L:2) |

^a^ Grade a reported from MRI scan on Fredericson scale [21, 22].

^b^ (L)Lines infantry recruits under take 14 weeks in phase one training, followed by 12 weeks in phase 2 for a total of 26 weeks in basic training. (P)Parachute regiment recruits undertake 14 weeks in phase one training followed by 14 weeks in phase 2 for a total of 28 weeks basic training.
